# Supplementary material for: Self-efficacy, self-esteem, and happiness in older adults: A cross-sectional study
Source: PLoS One. 2025 Mar 26;20(3):e0319269. doi: 10.1371/journal.pone.0319269 (PMC11940660; doi:10.1371/journal.pone.0319269)
Supplement: S1 Table — (PDF) [file pone.0319269.s001.pdf]

# S1 TABLE

| ***Variable*** | | ***N (%)*** | ***Happiness*** | **p-value*** |
|---|---|---|---|---|
| | | | ***Me ± SD*** | |
| **Gender** ** | Male | 204 (51) | 88.06 ± 17.95 | 0.428 |
| | Female | 196 (49) | 86.58 ± 19.52 | |
| **Marriage** ** | Married | 285 (71.3) | 88.71 ± 18.90 | 0.021 |
| | Single | 115 (28.8) | 83.93 ± 17.93 | |
| **status of income** *** | Pension | 57 | 95.85 ± 21.23 | 0.001 |
| | Saving | 51 | 85.16 ± 19.91 | |
| | Depend on spouse | 101 | 86.78 ± 18.25 | |
| | Depend on child | 144 | 83.95 ± 17.18 | |
| | self-employment | 47 | 90.91 ± 16.61 | |
| **Education**** | Under diploma | 299 | 85.28 ± 16.59 | 0.001 |
| | Diploma and higher | 101 | 93.43 ± 23.01 | |
| **History of smoking**** | No | 327 | 87.39 ± 18.93 | 0.902 |
| | Yes | 73 | 87.09 ± 17.90 | |
| **Physical activity** ** | No | 370 | 85.91 ± 17.71 | 0.001 |
| | Yes | 30 | 104.86 ± 21.58 | |
| **Having diseases** ** | No | 124 | 89.79 ± 19.88 | 0.079 |
