# Supplementary material for: Self-efficacy, self-esteem, and happiness in older adults: A cross-sectional study
Source: PLoS One. 2025 Mar 26;20(3):e0319269. doi: 10.1371/journal.pone.0319269 (PMC11940660; doi:10.1371/journal.pone.0319269)
Supplement: S2 Table — (DOCX) [file pone.0319269.s002.docx]

**S2 TABLE**

| **Variables** | 1 | 2 | 3 | Me ± SD |
| --- | --- | --- | --- | --- |
| **1= Self-efficacy** | 1 |  |  | 12.31 ± 7.92 |
| **2= Self-esteem** | 0.354 * | 1 |  | 18.48 ± 8.48 |
| **3= Happiness** | 0.747 * | 0.306 * | 1 | 22.78 ± 8.22 |
