# Supplementary material for: Self-efficacy, self-esteem, and happiness in older adults: A cross-sectional study
Source: PLoS One. 2025 Mar 26;20(3):e0319269. doi: 10.1371/journal.pone.0319269 (PMC11940660; doi:10.1371/journal.pone.0319269)
Supplement: S3 Table — (PDF) [file pone.0319269.s003.pdf]

S3 TABLE

Variables	ß	R2 change	F change	SE	P-value *
**Step 1**					
Age	-0.263*	0.153	8.83	0.130	0.001
Gender	0.015			1.860	
Marriage	0.017			2.046	
Income	-0.061			0.636	
Education level	0.068			2.170	
Smoking	0.020			2.403	
Physical activity	0.195*			3.463	
Having chronic disease	0.021			1.984	
**Step 2**					
Age	-0.110	0.43	204.02	0.093	< 0.001
Gender	0.020			1.305	
Marriage	0.043			1.446	
Income	-0.013			0.447	
Education level	0.006			1.536	
Smoking	-0.049			1.684	
Physical activity	0.098			2.458	
Having chronic disease	0.022			1.396	
**Self-esteem**	0.695*			0.065	
**Self-efficacy**	0.037			0.152	
Total R2	-	0.587	-	-	-
Adjusted R2	-	0.577	-	-	-
