# Supplementary material for: Self-efficacy, self-esteem, and happiness in older adults: A cross-sectional study
Source: PLoS One. 2025 Mar 26;20(3):e0319269. doi: 10.1371/journal.pone.0319269 (PMC11940660; doi:10.1371/journal.pone.0319269)
Supplement: S2 File — (DOCX) [file pone.0319269.s005.docx]

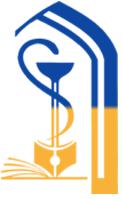


Sarab Medical Sciences Faculty


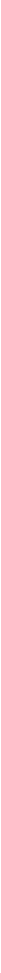

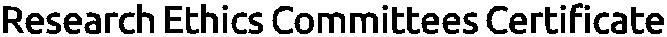


| Approval ID: |  |  |  |  |
| --- | --- | --- | --- | --- |
|  |  | Approval Date: | 2023-04-17 |  |
| Evaluated by: | Research Ethics Committees of Sarab Medical Sciences Faculty |  |  |  |
|  |  |  |  |  |
|  |  |  |  |  |
| Status: | Approved | |  |  |
|  |  | | |  |
| Approval | The project was found to be in accordance to the ethical principles and the national norms and | | |  |
| Statement: | standards for conducting Medical Research in Iran. | |  |  |
|  | Notice: | |  |  |
|  | Although the proposal has been approved by the Biomedical Research Ethics Committee, | | |  |
|  | meeting the professional and legal requirements is the sole responsibility of the PI and other | | |  |
|  | project collaborators. | |  |  |
|  | This certifcate is reliant on the proposal/documents received by this committee on | | |  |
|  | . The committee must be notifed by the PI as soon as the proposal/documents are | | |  |
|  | modifed. | |  |  |
|  |  | | |  |
| Proposal Title: | Factors affecting on happiness and explaining its barriers in the elderly: Examining the views of the | | |  |
|  | elderly, nurses and nursing students | |  |  |
|  |  | |  |  |
| Principal | : khalil maleki chulu | |  |  |
| Investigator: | : khmaleki444@gmail.com | |  |  |
|  |  |  |  |  |


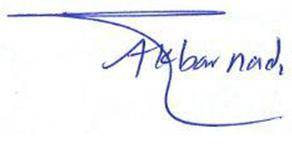

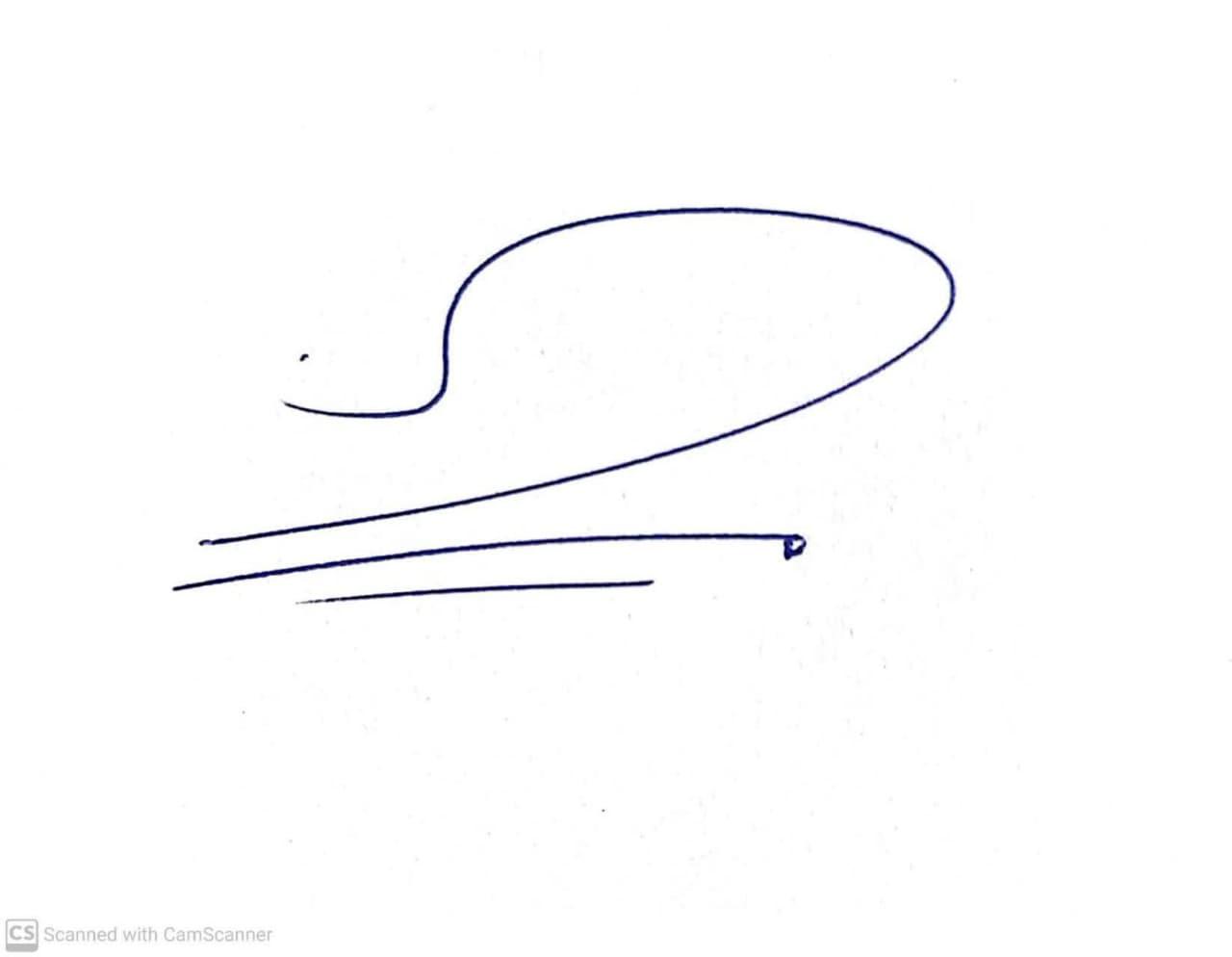

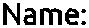

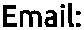

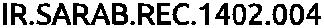

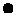

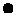

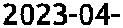

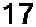


| Dr. Akbar Nadi | Dr. Nima Jadidian |
| --- | --- |
| Committee Director | Committee Secretary |
| Sarab Medical Sciences Faculty | Sarab Medical Sciences Faculty |

https://ethics.research.ac.ir/ProposalCertificateEn.php?id=٣٢٨٥١٣&Print=true&NoPrintHeader=true&NoPrintFooter=true&NoPrintPageBorder=tru… ١/١
