# Supplementary material for: Self-efficacy, self-esteem, and happiness in older adults: A cross-sectional study
Source: PLoS One. 2025 Mar 26;20(3):e0319269. doi: 10.1371/journal.pone.0319269 (PMC11940660; doi:10.1371/journal.pone.0319269)
Supplement: Attachment — Submitted filename: self-efficacy and self-esteem with happiness.docx [file pone.0319269.s006.docx]

Association self-efficacy and self-esteem with Happiness in elders: A cross-sectional study

**Dear [Dr/Aymen El Ashry ],**

I hope this message finds you well. I wanted to extend my sincere gratitude to you for sending me the article for review. I truly appreciate the opportunity to contribute to the process and offer my insights.

I've gone through the article and I have some constructive feedback that I believe could enhance the overall quality and impact of the piece.

- **Title**

Your research title is clear and informative, but it can be made more concise and engaging. Here are a few suggestions to refine and enhance the title:

- Happiness in the Elderly: The Role of Self-Efficacy and Self-Esteem in a Cross-Sectional Study
- The Relationship Between Self-Efficacy, Self-Esteem, and Happiness in Older Adults: A Cross-Sectional study
- Exploring the Link Between Self-Efficacy, Self-Esteem, and Happiness Among Elders: A Cross-Sectional Study
- **Abstract**
- Expand the background to briefly explain the theoretical or empirical basis for focusing on self-efficacy and self-esteem in relation to happiness in the elderly.
- Mention the specific tools or scales used to measure self-efficacy, self-esteem, and happiness.
- Conclusion: The conclusion is somewhat vague regarding how to design programs to increase self-efficacy and self-esteem. Suggest specific types of programs or interventions that could be implemented to enhance self-efficacy and self-esteem among the elderly.
- **Introduction**
- **Lack of Specificity**: The phrase "advancements in medical care, public health, and socio-economic progress" is vague. Providing specific examples or types of advancements would enhance clarity
- **Redundancy**: The idea that life expectancy has increased leading to a larger elderly population is repeated in the statistics that follow. Combining these statements could make the paragraph more concise.
- **Abrupt Transition**: The transition from demographic statistics to the discussion on shifting health priorities is abrupt. A connecting sentence would improve the flow.
- **Insufficient Explanation**: The claim that "mental health is even more crucial than physical health for older adults" is a significant assertion that needs more context or examples to substantiate why mental health is deemed more critical.
- **Citation Placement**: The placement of citations could be more precise. For example, placing the citation immediately after the relevant statistics (e.g., after "In 2020, 13.5% of the global population was 60 years or older (2).
- **Methods**
- **Clarity in Sampling Formula**: The sample size formula is presented as a mathematical equation but isn't clearly formatted for readability. Additionally, there is no explanation of the terms used in the formula.
- **Inclusion and Exclusion Criteria**: The inclusion criteria mention "diagnosed with hypertension," but it's unclear why this specific condition was chosen or how it relates to the study's focus on happiness, self-efficacy, and self-esteem.
- **Sampling Method Description**: The term "multi-state cluster random method" is used, which might be confusing. It should be clarified if this means multi-stage cluster sampling and how exactly the clusters and participants were selected.
- **Data Collection Process**: The description of the data collection process is brief and lacks details on how the interviews were conducted, how data accuracy was ensured, and if any steps were taken to minimize bias.
- **Interviewer Training**: There is no mention of how the two educated students who conducted the interviews were trained, which could affect the consistency and reliability of data collection.
- **Validation of Instruments**: While the internal consistency of the instruments is mentioned, more information on the process of validation, especially in the context of the local population, would strengthen the methodology.
- **Handling Missing Data**: There is no mention of how missing data were handled, which is important for ensuring the validity of the results.
- **Ethical Considerations**: There is no explicit mention of ethical approval or the process followed to ensure ethical standards were maintained during the study.
- **Statistical Software Version**: The mention of "SPSS 21 software" could be updated to ensure readers know the analysis was done using a relevant and recent version of the software.
- **Details on Hierarchical Regression**: The explanation of hierarchical regression could be more detailed, particularly on why this method was chosen and how the blocks were determined.
- **Discussion**

The discussion section has several weak points that need addressing. Redundancies, such as repeating the association of self-efficacy and self-esteem with happiness, should be streamlined for clarity. The analysis lacks depth, providing a summary of results without a deeper exploration of underlying reasons. Terms like self-confidence are inconsistently used and not clearly defined, potentially confusing readers. There is also an over-reliance on correlations without discussing potential causation or other influencing factors. Practical implications are vague, with no specific recommendations for interventions. Transitions between points are abrupt, making the discussion difficult to follow. Additionally, the discussion does not sufficiently compare findings with existing literature or address study limitations, such as the cross-sectional design and generalizability. Finally, some statistical results are repeated without adding new insights, unnecessarily lengthening the discussion.

- **Please address implications for nursing research, education and practice**

**Once again, thank you for entrusting me with this task. I look forward to our continued collaboration and to seeing the final version of the article.**

**Warm regards,**

Shaimaa Mohamed Amin
