# Supplementary material for: Self-efficacy, self-esteem, and happiness in older adults: A cross-sectional study
Source: PLoS One. 2025 Mar 26;20(3):e0319269. doi: 10.1371/journal.pone.0319269 (PMC11940660; doi:10.1371/journal.pone.0319269)
Supplement: Attachment — Submitted filename: Response to reviewers.docx [file pone.0319269.s009.docx]

**POINT-BY-POINT RESPONSE FORM**

**Title: Response to Reviewer Comments on Manuscript**

To the Editor of Plos One : Dr Ayman Mohamed El-Ashry

Manuscript : PONE-D-24-19287

Dear Editor,

On behalf of my co-authors, I, Khalil Maleki Chollou, would like to express our sincere gratitude to you and the reviewers for the thorough review and valuable feedback on our manuscript . Your comments will greatly help us improve the quality of our paper.

In this letter, we intend to address each of the reviewers' comments and suggestions in detail.

| **Suggestion, Question,**  **or Comment from the**  **Editor** | **Author’s Response** | **Change in the Manuscript** |
| --- | --- | --- |
| Refined Research Title Suggestions:  Happiness in the Elderly: Self-Efficacy and Self-Esteem in a Cross-Sectional Study  Self-Efficacy, Self-Esteem, and Happiness in Older Adults: A Cross-Sectional Study  Linking Self-Efficacy, Self-Esteem, and Happiness in Elders: A Cross-Sectional Study | We truly appreciate the Editor’s suggestion, and we recognize that it would improve the revised version of the manuscript. | Change of Manuscript Title from “Association self-efficacy and self-esteem with Happiness in elders: A cross-sectional study” to “Linking Self-Efficacy, Self-Esteem and Happiness in Older People: A Cross-Sectional Study” |
| Abstract:  Background: Briefly outline theoretical or empirical foundations for studying self-efficacy and self-esteem in relation to elderly happiness.  Measurement Tools: Specify the scales or tools used to assess self-efficacy, self-esteem, and happiness.  Conclusion: Offer specific suggestions for programs or interventions to enhance self-efficacy and self-esteem among the elderly. | We have meticulously reviewed your feedback and have implemented substantial revisions to the abstract, enhancing its clarity, conciseness, and overall impact. Your valuable insights have been instrumental in refining the abstract to accurately reflect the core findings and significance of the research | In line 33-35, the **Background** was modified with the following paragraph:  “Happiness is crucial for well-being in older people, but it can be challenged by various health issues. Self-efficacy, the belief in one's ability to manage challenges, and self-esteem, positive self-regard, are theorized to play significant roles in promoting happiness in this population.”  In line 39-45, the **Methods** was modified with the following paragraph:  “A cross-sectional study was conducted with 400 individuals aged 60 years or older who visited health centers in Sarab, Iran, from April to June 2023. Data were collected using valid and reliable instruments, including the Oxford Happiness Questionnaire, Sherer’s Self-Efficacy Scale, and the Rosenberg Self-Esteem Scale. To analyze the data, bivariate comparisons of quantitative variables were performed using independent samples t-tests and one-way ANOVA. Additionally, hierarchical regression analysis was conducted on happiness using two distinct sets of independent variables.”  In line 56-61, the **Conclusion** was modified with the following paragraph:  “To enhance happiness in elderly populations, healthcare centers frequented by this demographic could implement programs designed to bolster self-efficacy and self-esteem. Examples include lifelong learning initiatives, support groups, and art therapy interventions. The use of effectiveness of such interventions in promoting happiness among older adults can promote the happiness in elderly people warrants further investigation in future studies..” |
| Introduction:  Specificity: Detail specific advancements in medical care, public health, and socio-economic progress.  Redundancy: Combine life expectancy increase statements for conciseness.  Transition: Smooth the transition from demographic statistics to health priorities.  Explanation: Provide context or examples for the assertion that mental health is crucial for older adults.  Citation: Place citations immediately after relevant statistics for clarity. | We have meticulously reviewed your feedback and have implemented substantial revisions to the Introduction, enhancing its clarity, conciseness, and overall impact. Your valuable insights have been instrumental in refining the Introduction to accurately reflect the core findings and significance of the research | Significant revisions were made throughout the introduction to enhance clarity and conciseness. |
| Sampling Formula: Clearly format and explain terms in the sample size formula. | Thank you for your valuable feedback. We have revised the description of the sample size calculation to provide a clearer and more detailed explanation. | In line 134-141, was modified. |
| Criteria Justification: Explain the choice of hypertension as an inclusion criterion and its relation to the study's focus. | Thank you for your insightful comment. We appreciate the opportunity to clarify this point. The study was designed to include elderly participants regardless of their hypertension status, as we aimed to capture a broad spectrum of health conditions within the elderly population. The mention of hypertension as an inclusion criterion was not intended to imply exclusivity, but rather to acknowledge the commonality of such conditions in the target demographic. In practice, the study included both individuals with and without hypertension to ensure a comprehensive analysis. We have revised the manuscript to more accurately reflect this approach. |  |
| Sampling Method: Clarify the term "multi-state cluster random method" and describe participant selection. | Thank you for your comment. We have clarified the sampling method and participant selection process in the revised manuscript | In line 146-151, was modified. |
| Data Collection: Elaborate on the data collection process, interviewer training, and steps to ensure data accuracy and minimize bias. | Thank you for your comment. We have expanded on the data collection process, including interviewer training and measures taken to ensure data accuracy and minimize bias. | In line 154-165, was modified. |
| Instrument Validation: Detail the validation process for instruments in the local context. | Thank you for your comment. All necessary revisions have been made to ensure that the validation process is detailed and specific to the local context. | In line 171-204, was modified. |
| Missing Data: Describe how missing data were handled. | Thank you for your comment. As we employed a face-to-face interview technique for data collection, every effort was made to ensure that all data were collected comprehensively. Due to the direct interaction between interviewers and participants, no data were missing, and all questionnaires were fully completed. | In line 239-241, was added |
| Ethical Considerations: Mention ethical approval and adherence to ethical standards. | Thank you for your comment.Done | In line 126-128, was modified |
| Software Version: Update or confirm the statistical software version used. | Thank you for your comment. The statistical analyses were performed using SPSS version 26. We have updated the manuscript to reflect the correct version of the software used. |  |
| Hierarchical Regression: Provide a detailed rationale for choosing hierarchical regression and block determination. | Thank you for your insightful comment. Hierarchical regression was chosen for this study because it allows for the assessment of the incremental value of additional predictors in explaining the variance in happiness. By entering variables in blocks, we could observe how much additional variance was explained by including psychological factors such as self-efficacy and self-esteem after accounting for basic demographic characteristics.  Block 1 included demographic variables because these factors are foundational and often influence psychological outcomes. In Block 2, self-efficacy and self-esteem were added to examine whether these psychological factors provide a significant contribution to the explanation of happiness, beyond what is explained by demographic variables alone. The change in adjusted R² between these blocks provided insight into the unique contribution of these psychological variables.  We have revised the manuscript to incorporate this rationale more explicitly and to clarify the determination of the blocks. | In line 219-229, was modified |
| Discussion:  Streamlining: Remove redundancies, particularly regarding the association of self-efficacy and self-esteem with happiness.  Depth: Deepen the analysis by exploring underlying reasons for results.  Term Consistency: Define and consistently use terms like self-confidence.  Correlations and Causation: Discuss potential causation and other influencing factors beyond correlations.  Practical Implications: Offer specific recommendations for interventions.  Transitions: Improve transitions between points for better readability.  Comparison with Literature: Compare findings with existing literature and address study limitations, including cross-sectional design and generalizability.  Statistical Results: Avoid unnecessary repetition of statistical results. | All the comments were made point-by-point and marked by track changes and highlighting in the revision file of the manuscript. |  |
|  |  |  |
| **Suggestion, Question,**  **or Comment from**  **Reviewer #1** | **Author’s Response** | **Change in the Manuscript** |
| Your research title is clear and informative, but it can be made more concise and engaging. Here are a few suggestions to refine and enhance the title:  • Happiness in the Elderly: The Role of Self-Efficacy and Self-Esteem in a Cross-Sectional Study  • The Relationship Between Self-Efficacy, Self-Esteem, and Happiness in Older Adults: A Cross-Sectional study  • Exploring the Link Between Self-Efficacy, Self-Esteem, and Happiness Among Elders: A Cross-Sectional Study | We truly appreciate the **Reviewer #1** suggestion, and we recognize that it would improve the revised version of the manuscript. | Change of Manuscript Title from “Association self-efficacy and self-esteem with Happiness in elders: A cross-sectional study” to “Linking Self-Efficacy, Self-Esteem and Happiness in Older People: A Cross-Sectional Study” |
| Abstract  • Expand the background to briefly explain the theoretical or empirical basis for focusing on self-efficacy and self-esteem in relation to happiness in the elderly.  • Mention the specific tools or scales used to measure self-efficacy, self-esteem, and happiness.  • Conclusion: The conclusion is somewhat vague regarding how to design programs to increase self-efficacy and self-esteem. Suggest specific types of programs or interventions that could be implemented to enhance self-efficacy and self-esteem among the elderly. | We have meticulously reviewed your feedback and have implemented substantial revisions to the abstract, enhancing its clarity, conciseness, and overall impact. Your valuable insights have been instrumental in refining the abstract to accurately reflect the core findings and significance of the research | In line 33-35, the **Background** was modified with the following paragraph:  “Happiness is crucial for well-being in older people, but it can be challenged by various health issues. Self-efficacy, the belief in one's ability to manage challenges, and self-esteem, positive self-regard, are theorized to play significant roles in promoting happiness in this population.”  In line 39-45, the **Methods** was modified with the following paragraph:  “A cross-sectional study was conducted with 400 individuals aged 60 years or older who visited health centers in Sarab, Iran, from April to June 2023. Data were collected using valid and reliable instruments, including the Oxford Happiness Questionnaire, Sherer’s Self-Efficacy Scale, and the Rosenberg Self-Esteem Scale. To analyze the data, bivariate comparisons of quantitative variables were performed using independent samples t-tests and one-way ANOVA. Additionally, hierarchical regression analysis was conducted on happiness using two distinct sets of independent variables.”  In line 56-61, the **Conclusion** was modified with the following paragraph:  “To enhance happiness in elderly populations, healthcare centers frequented by this demographic could implement programs designed to bolster self-efficacy and self-esteem. Examples include lifelong learning initiatives, support groups, and art therapy interventions. The use of effectiveness of such interventions in promoting happiness among older adults can promote the happiness in elderly people warrants further investigation in future studies..” |
| Lack of Specificity: The phrase "advancements in medical care, public health, and socio-economic progress" is vague. Providing specific examples or types of advancements would enhance clarity  Redundancy: The idea that life expectancy has increased leading to a larger elderly population is repeated in the statistics that follow. Combining these statements could make the paragraph more concise. | Thank you for this valuable suggestion. In response,we have revised the manuscript to include specific examples of advancements in these areas. | In line 74-77, was modified with the following paragraph:  “Nowadays, with advancements in medical care and public health system resulting in the control and treatment of diseases, as well as social and economic progress that have led to the end of extensive wars, improvements in living conditions and the revolutions in the agriculture, increased the lifespan of humans” |
| Abrupt Transition: The transition from demographic statistics to the discussion on shifting health priorities is abrupt. A connecting sentence would improve the flow. | Thank you for pointing out this issue. We have added a connecting sentence to bridge the transition between the demographic statistics and the discussion on shifting health priorities. This addition aims to enhance the flow and coherence of the text. The revised section now provides a smoother transition and improved continuity. The changes can be found in the updated manuscript | In line 81-83, was added with the following paragraph:  “This demographic transition poses significant challenges for healthcare systems, which must adapt to meet the needs of older adults . They need to find effective strategies to extend health care and to respond to the needs of older adults ” |
| Insufficient Explanation: The claim that "mental health is even more crucial than physical health for older adults" is a significant assertion that needs more context or examples to substantiate why mental health is deemed more critical. | Thank you for highlighting this important point. We have revised the sentence to provide additional context and examples to better substantiate the assertion that mental health is crucial for older adults. The revised sentence now includes specific evidence and references to support this claim. | In line 88-89, was added with the following paragraph:  “According to Levinson and Kaplan, self-rated mental health is more important than self-rated physical health in predicting overall self-rated health ” |
| Citation Placement: The placement of citations could be more precise. For example, placing the citation immediately after the relevant statistics (e.g., after "In 2020, 13.5% of the global population was 60 years or older (2). | Thank you for your insightful comment, reference number 2 belongs to the whole sentence, that is: According to statistics reported in 2020, 13.5 percent of the world's population was 60 years old or older and it is estimated that by 2050, one in every five people will be older adults |  |
| Clarity in Sampling Formula: The sample size formula is presented as a mathematical equation but isn't clearly formatted for readability. Additionally, there is no explanation of the terms used in the formula. | Thank you for your valuable feedback. We have revised the description of the sample size calculation to provide a clearer and more detailed explanation. | In line 134-141, was modified. |
| Inclusion and Exclusion Criteria: The inclusion criteria mention "diagnosed with hypertension," but it's unclear why this specific condition was chosen or how it relates to the study's focus on happiness, self-efficacy, and self-esteem. | Thank you for your insightful comment. We appreciate the opportunity to clarify this point. The study was designed to include elderly participants regardless of their hypertension status, as we aimed to capture a broad spectrum of health conditions within the elderly population. The mention of hypertension as an inclusion criterion was not intended to imply exclusivity, but rather to acknowledge the commonality of such conditions in the target demographic. In practice, the study included both individuals with and without hypertension to ensure a comprehensive analysis. We have revised the manuscript to more accurately reflect this approach. |  |
| Sampling Method Description: The term "multi-state cluster random method" is used, which might be confusing. It should be clarified if this means multi-stage cluster sampling and how exactly the clusters and participants were selected. | Thank you for your comment. We have clarified the sampling method and participant selection process in the revised manuscript | In line 146-151, was modified. |
| Data Collection Process: The description of the data collection process is brief and lacks details on how the interviews were conducted, how data accuracy was ensured, and if any steps were taken to minimize bias.  Interviewer Training: There is no mention of how the two educated students who conducted the interviews were trained, which could affect the consistency and reliability of data collection. | Thank you for your comment. We have expanded on the data collection process, including interviewer training and measures taken to ensure data accuracy and minimize bias. | In line 154-165, was modified. |
| Validation of Instruments: While the internal consistency of the instruments is mentioned, more information on the process of validation, especially in the context of the local population, would strengthen the methodology. | Thank you for your comment. All necessary revisions have been made to ensure that the validation process is detailed and specific to the local context. | In line 171-204, was modified. |
| Handling Missing Data: There is no mention of how missing data were handled, which is important for ensuring the validity of the results. | Thank you for your comment. As we employed a face-to-face interview technique for data collection, every effort was made to ensure that all data were collected comprehensively. Due to the direct interaction between interviewers and participants, no data were missing, and all questionnaires were fully completed. | In line 239-241, was added |
| Ethical Considerations: There is no explicit mention of ethical approval or the process followed to ensure ethical standards were maintained during the study. | Thank you for your comment.Done | In line 126-128, was modified |
| Statistical Software Version: The mention of "SPSS 21 software" could be updated to ensure readers know the analysis was done using a relevant and recent version of the software. | Thank you for your comment. The statistical analyses were performed using SPSS version 26. We have updated the manuscript to reflect the correct version of the software used. |  |
| Details on Hierarchical Regression: The explanation of hierarchical regression could be more detailed, particularly on why this method was chosen and how the blocks were determined. | Thank you for your insightful comment. Hierarchical regression was chosen for this study because it allows for the assessment of the incremental value of additional predictors in explaining the variance in happiness. By entering variables in blocks, we could observe how much additional variance was explained by including psychological factors such as self-efficacy and self-esteem after accounting for basic demographic characteristics.  Block 1 included demographic variables because these factors are foundational and often influence psychological outcomes. In Block 2, self-efficacy and self-esteem were added to examine whether these psychological factors provide a significant contribution to the explanation of happiness, beyond what is explained by demographic variables alone. The change in adjusted R² between these blocks provided insight into the unique contribution of these psychological variables.  We have revised the manuscript to incorporate this rationale more explicitly and to clarify the determination of the blocks. | In line 219-229, was modified |
| Discussion  The discussion section has several weak points that need addressing. Redundancies, such as repeating the association of self-efficacy and self-esteem with happiness, should be streamlined for clarity. The analysis lacks depth, providing a summary of results without a deeper exploration of underlying reasons. Terms like self-confidence are inconsistently used and not clearly defined, potentially confusing readers. There is also an over-reliance on correlations without discussing potential causation or other influencing factors. Practical implications are vague, with no specific recommendations for interventions. Transitions between points are abrupt, making the discussion difficult to follow. Additionally, the discussion does not sufficiently compare findings with existing literature or address study limitations, such as the cross-sectional design and generalizability. Finally, some statistical results are repeated without adding new insights, unnecessarily lengthening the discussion. | All the comments were made point-by-point and marked by track changes and highlighting in the revision file of the manuscript. |  |
|  |  |  |
| **Suggestion, Question,**  **or Comment from**  **Reviewer #2** | **Author’s Response** | **Change in the Manuscript** |
| It is better to use term "older people “instead of "elderly", “elders" | Thank you for your suggestion. We have made the recommended changes, replacing the terms "elderly" and "elders" with "older people" throughout the manuscript. |  |
| Put more information in the abstract, method section. | Thank you for your valuable feedback. We have revised the manuscript to include more detailed information in both the abstract and the method section |  |
| In view of the high prevalence of chronic diseases such as high blood pressure during old age, how many were the total number of elderly people without high blood pressure in Sarab city, out of which 400 samples were randomly selected? How to select and access samples should also be mentioned.  Please explain why the authors chose people without blood pressure? | Thank you for your question. In this study, participants were selected regardless of their hypertension status to ensure a comprehensive representation of the elderly population in Sarab city. The study did not specifically exclude individuals with hypertension, as the aim was to include a broad range of health conditions typical of the elderly demographic. Therefore, both individuals with and without high blood pressure were randomly selected from the total elderly population using health records from the local health centers. This approach allowed for a more inclusive and representative sample. We have clarified the selection process and criteria in the revised manuscript. |  |
| It is written in the working method section that the questionnaires were completed by the questioner. In this case, please explain what measures were used to avoid interfering with the questioner's opinion in completing the information? | Thank you for your comment. In the revised version of the manuscript, we have provided detailed explanations regarding the measures taken to avoid any interference from the interviewer's opinions during the completion of the questionnaires. | In line 154-165, was modified. |
| In the demographic information section, what type of disease did you mean by disease? | Thank you for your comment. In the demographic information section, the term "disease" encompasses a range of chronic conditions commonly found among the elderly. Specifically, the study included participants with conditions such as cancer, chronic respiratory diseases, cardiovascular diseases, and diabetes. |  |
| Regarding the happiness tool, the mentioned reference is related to the research done in India, please put the reference related to the Oxford Happiness Questionnaire first, and then the validity and reliability of the questionnaire in Persian and English version, along with mentioning the references to be mentioned separately. Also, write the minimum and maximum score of this questionnaire. If the validity and reliability of this questionnaire has been done in your article, please mention it. Also explain how to interpret the score obtained from the questionnaire. | Thank you for your feedback. All possible changes have been made to improve the manuscript. |  |
| In relation to the self-efficacy questionnaire, the reference mentioned does not match what was referred. Please refer to the original author of the questionnaire. Also, Cronbach's alpha should be written both in the main study and in the study conducted in Iran. Cronbach's alpha should be written instead of the term alpha value. | Thank you for your feedback. All possible changes have been made to improve the manuscript. |  |
| Regarding the self-esteem scale questionnaire, please add the original reference and write its psychometric description. | Thank you for your feedback. All possible changes have been made to improve the manuscript. |  |
| Please specify the independent and dependent variables of this research. | Thank you for your comment. In the revised version of the manuscript, we have provided clear explanations regarding the independent and dependent variables of the research. Specifically, the independent variables include demographic characteristics (such as age, gender, marital status, education, and income), self-efficacy, and self-esteem. The dependent variable in this study is happiness. | In line 149-160, was added. |
| In TABLE 1 regarding the marital status, only two groups, single and married, are mentioned, while there are also divorced people and deceased spouses. What has been done about these people? | Thank you for your comment. In the analysis, individuals who were divorced or had deceased spouses were categorized under the "single" group for the purpose of simplicity and statistical analysis. |  |
| Instead of income, write the status of income. | Thank you for your comment.Done |  |
| Why is education divided into two categories below diploma and above diploma?  If it seems that the classification of elderly people in the sub-diploma category with more classes is more important. Because most of the elderly do not have a high level of education. Also, did you have an illiterate person in this study? This is despite the fact that you mentioned in the findings section that 43% of people were illiterate. | Thank you for your valuable feedback. We appreciate your observation regarding the classification of education levels. The decision to divide education into two categories (below diploma and above diploma) was made to simplify the analysis and to make a clear distinction between those with higher and lower levels of education.  Regarding the presence of illiterate participants, we confirm that 43% of the participants in our study were indeed illiterate. These individuals were included in the below diploma category for the purposes of analysis. |  |
| In the discussion section, the statistically significant type should be mentioned and explained. You are the only one who mentions that there is a statistically significant association. You should write, for example, with increasing physical activity, the level of happiness increased and... | All the comments were made point-by-point and marked by track changes and highlighting in the revision file of the manuscript. |  |

With the changes outlined in this letter, we are confident that the manuscript  is scientifically richer and of higher quality.

We thank you and the reviewers for your patience and thorough review of this manuscript.

Sincerely,

Khalil Maleki Chollou
