# Supplementary material for: Self-efficacy, self-esteem, and happiness in older adults: A cross-sectional study
Source: PLoS One. 2025 Mar 26;20(3):e0319269. doi: 10.1371/journal.pone.0319269 (PMC11940660; doi:10.1371/journal.pone.0319269)
Supplement: Attachment — Submitted filename: Reviewer report.docx [file pone.0319269.s008.docx]

**Review comments**

| **Title** | The current title is clear, but it could be more concise while still capturing the essence of the study. For example:  "Self-Efficacy, Self-Esteem, and Happiness in Older Adults: A Cross-Sectional Study" |
| --- | --- |
| **Abstract** | **Background:** The abstract is generally clear, but adding a more explicit statement on the research gap would strengthen it.  **Results:** Include specific quantitative data to reinforce your claims. For example, when mentioning the hierarchical multiple regression analysis, include the exact values for the coefficients or percentages of variance explained.  **Conclusion:** Your conclusion mentions interventions, but this could be more specific. Instead of broadly suggesting “support groups,” you could suggest types of interventions that have been proven to work, like "skill-building workshops aimed at enhancing self-efficacy." |
| **Introduction** | **Specificity:** Be more specific about the advancements in medical care and public health mentioned. Providing examples or citations can give more context. For instance, highlight advancements in particular areas like preventive medicine or technology that extend health span.  **Research Gap:** it would be beneficial to expand on why Iranian elderly rank low in happiness compared to global counterparts, perhaps by discussing specific cultural or socioeconomic factors unique to Iran.  **Clarity:** Some parts, especially around the discussion of cognitive factors feel a bit abstract. You can ground these concepts by referring to specific studies or providing more concrete examples. |
| **Methods** | **Sampling Method:** The term "multi-stage cluster sampling" is mentioned, but the process could be better clarified. You might want to add a sentence or two explaining why this method was chosen and how it benefits the study. You could elaborate on how clusters were selected (e.g., health centers or regions), and how this sampling ensures representativeness.  **Inclusion Criteria:** It's great that you have clear inclusion/exclusion criteria, but it’s important to explain why participants with psychiatric disorders were excluded. What impact would their inclusion have on the study’s outcomes?  **Data Collection:** Adding a brief explanation about the measures taken to ensure that interviewers’ bias was minimized (e.g., through standardized scripts or blind interviews) could strengthen the validity of your methods. |
| **Results** | **Interpretation of Statistical Data:** In Table 3, the values could be interpreted more clearly. You could explain how the changes in R² (e.g., from 0.153 to 0.587) reflect the significant role of self-efficacy and self-esteem beyond demographic factors.  **Detailing Key Findings:** For the significant predictors, such as self-efficacy and self-esteem, a brief explanation on why they contribute so heavily to happiness in your population could provide a deeper understanding of your results. |
| **Discussion** | **Causal Inferences:** The discussion seems to make causal interpretations from a cross-sectional study. Since your study is correlational, emphasize that while relationships were found, causality cannot be inferred. Adding a statement to this effect in the discussion and limitations will strengthen the scientific rigor of your work.  **Comparative Analysis:** Compare your findings more explicitly with studies in different cultural or geographical settings. You cite many studies from other countries (e.g., Turkey, Thailand), but a more nuanced comparison of how these findings align with or differ from your results will add depth.  **Mechanisms of Influence:** In lines 266-268, you mention that increasing self-efficacy and self-esteem can enhance happiness. Discuss potential mechanisms for this influence. For instance, is it because of improved coping strategies, greater social participation, or reduced psychological distress?  **Interventions:** In lines 305-307, be more specific about the types of community-based programs you recommend. For example, "Programs incorporating cognitive-behavioral therapy or mindfulness training could effectively boost self-efficacy and self-esteem." |
| **Limitations** | **Expanded Limitations:** You mention that the study’s cross-sectional design limits your ability to draw causal conclusions. It would be beneficial to mention other limitations, such as reliance on self-reported measures, which can introduce bias. Also, acknowledge any potential biases in sampling (e.g., if the study underrepresents a certain subgroup of the elderly population in Sarab).  **Generalizability:** The generalizability of your results outside the Iranian context could be addressed. You may want to elaborate on how cultural differences might affect self-efficacy, self-esteem, and happiness relationships in other contexts. |
| **Conclusion** | **Action-Oriented Suggestions:** Your conclusion should not only summarize the findings but also provide clearer actionable recommendations for healthcare providers and policymakers. Rather than just suggesting “preventive and supportive actions,” you could specify programs that could be implemented, such as "targeted cognitive-behavioral programs aimed at boosting self-efficacy and emotional regulation in older adults." |
| **Language and Formatting** | **Clarity and Consistency:** The manuscript can be improved by revising some repetitive phrases and ensuring consistency in terms (e.g., consistently using either "older adults" or "elderly" rather than switching between them). Also, ensure that all abbreviations are defined the first time they are used.  **Grammar and Flow:** In some sections, the flow of ideas is somewhat disrupted by repetitive transitions (e.g., lines 235-236). Try to vary sentence structure and reduce redundancy to improve readability. |
| **Novelty** | Emphasize the unique contributions of your research. For example, highlight how your study provides new insights into an under-researched population (Iranian elderly) in the context of self-efficacy, self-esteem, and happiness. Stress this in both the introduction and conclusion to reinforce the study’s value. |
