# Supplementary material for: Self-efficacy, self-esteem, and happiness in older adults: A cross-sectional study
Source: PLoS One. 2025 Mar 26;20(3):e0319269. doi: 10.1371/journal.pone.0319269 (PMC11940660; doi:10.1371/journal.pone.0319269)
Supplement: Attachment — Submitted filename: Response_to_reviewers_auresp_2.docx [file pone.0319269.s010.docx]

**POINT-BY-POINT RESPONSE FORM**

**Title: Response to Reviewer Comments on Manuscript**

To the Editor of Plos One : Dr Ayman Mohamed El-Ashry

Manuscript : PONE-D-24-19287

Dear Editor,

On behalf of my co-authors, I, Khalil Maleki Chollou, would like to express our sincere gratitude to you and the reviewers for the thorough review and valuable feedback on our manuscript . Your comments will greatly help us improve the quality of our paper.

In this letter, we intend to address each of the reviewers' comments and suggestions in detail.

| **Suggestion, Question,**  **or Comment from the**  **Reviewer #3** | **Author’s Response** | **Change in the Manuscript** |
| --- | --- | --- |
| The current title is clear, but it could be more concise while still capturing the essence of the study. For example:  "Self-Efficacy, Self-Esteem, and Happiness in Older Adults: A Cross-Sectional Study" | We truly appreciate the Reviewer #3’s suggestion, and we recognize that it would improve the revised version of the manuscript. | Change of Manuscript Title from “Linking Self-Efficacy, Self-Esteem and Happiness in Older People: A Cross-Sectional Study” to “Self-Efficacy, Self-Esteem, and Happiness in Older Adults: A Cross-Sectional Study” |
| **Abstract Background:** The abstract is generally clear, but adding a more explicit statement on the research gap would strengthen it. | Thank you for this valuable suggestion. We have revised the Background section of the abstract to explicitly state the research gap by highlighting the lack of studies examining the combined effect of self-efficacy and self-esteem on happiness in elderly populations. | This can be found in lines 29–34 of the revised abstract. |
| **Abstract Results:** Include specific quantitative data to reinforce your claims. For example, when mentioning the hierarchical multiple regression analysis, include the exact values for the coefficients or percentages of variance explained. | Thank you for this valuable suggestion. We have revised the Results section of the abstract. | This can be found in lines 42–48 of the revised abstract. |
| **Abstract Conclusion:** Your conclusion mentions interventions, but this could be more specific. Instead of broadly suggesting “support groups,” you could suggest types of interventions that have been proven to work, like "skill-building workshops aimed at enhancing self-efficacy." | We appreciate this suggestion to provide more specific recommendations. We have revised the Conclusion to include more targeted interventions, such as "skill-building workshops" and "art therapy programs." | These changes are now reflected in lines 49–56 of the revised abstract. |
| **Introduction Specificity:** Be more specific about the advancements in medical care and public health mentioned. Providing examples or citations can give more context. For instance, highlight advancements in particular areas like preventive medicine or technology that extend health span. | we have added specific examples of advancements in medical care, including improvements in preventive medicine and technological innovations like telemedicine and wearable health monitors, to provide clearer context. | These changes are now reflected in lines 60–65 of the revised Introduction. |
| **Introduction** **Research Gap**: it would be beneficial to expand on why Iranian elderly rank low in happiness compared to global counterparts, perhaps by discussing specific cultural or socioeconomic factors unique to Iran. | We have expanded the discussion on why Iranian seniors rank low in happiness by including cultural and socioeconomic factors unique to Iran, such as economic instability and limited social support networks. | This can be found in lines 102–106 of the revised Introduction |
| **Introduction** **Clarity**: Some parts, especially around the discussion of cognitive factors feel a bit abstract. You can ground these concepts by referring to specific studies or providing more concrete examples. | We truly appreciate the Reviewer #3’s suggestion, and we recognize that it would improve the revised version of the manuscript. |  |
| **Methods Sampling Method**: The term "multi-stage cluster sampling" is mentioned, but the process could be better clarified. You might want to add a sentence or two explaining why this method was chosen and how it benefits the study. You could elaborate on how clusters were selected (e.g., health centers or regions), and how this sampling ensures representativeness. | Thank you for your insightful comments regarding the sampling method used in our study. In response to your suggestion for clarification, I have revised the description of the sampling method. | These changes are now reflected in lines 133–141 of the revised Methods. |
| **Methods Inclusion Criteria**: It's great that you have clear inclusion/exclusion criteria, but it’s important to explain why participants with psychiatric disorders were excluded. What impact would their inclusion have on the study’s outcomes? | Thank you for your insightful comment regarding the inclusion criteria for participants in our study. We appreciate your attention to detail and the importance of clearly explaining our rationale for excluding individuals with psychiatric disorders.  In this study, participants were individuals aged 60 years or older, free from psychiatric disorders, and willing to sign a consent form to participate. The exclusion of individuals with psychiatric disorders was implemented for several reasons:  Minimizing Confounding Variables: Individuals with psychiatric disorders may experience different levels of happiness, self-efficacy, and self-esteem compared to those without such disorders. Their inclusion could introduce confounding variables that might skew the results and complicate the interpretation of the relationships between self-efficacy, self-esteem, and happiness.  Focus on Psychological Constructs: The primary focus of our study is to investigate the relationships between self-efficacy, self-esteem, and happiness in a generally healthy elderly population. By excluding individuals with psychiatric disorders, we aimed to ensure that our findings accurately reflect the influence of these psychological constructs in a population where other mental health factors do not significantly interfere.  Enhancing Validity: By creating a more homogeneous sample, we enhance the validity and reliability of our findings. This allows us to draw more precise conclusions about the associations we are investigating and to better inform interventions aimed at improving the well-being of older adults.  We hope that this explanation addresses your concerns and clarifies the rationale behind our inclusion and exclusion criteria. |  |
| **Methods Data Collection**: Adding a brief explanation about the measures taken to ensure that interviewers’ bias was minimized (e.g., through standardized scripts or blind interviews) could strengthen the validity of your methods. | Thank you for this insightful comment. To address your concern, I have added further explanations regarding the measures implemented to minimize interviewer bias in the data collection section. | These changes are now reflected in lines 150–157 of the revised Methods. |
| **Results Interpretation of Statistical Data:** In Table 3, the values could be interpreted more clearly. You could explain how the changes in R² (e.g., from 0.153 to 0.587) reflect the significant role of self-efficacy and self-esteem beyond demographic factors. | We appreciate the reviewer’s insightful suggestion regarding the interpretation of Table 3. We have revised the Results section to provide a clearer explanation of the changes in R². Specifically, we now emphasize that the initial model with demographic factors explained 15.3% of the variation in happiness, indicating their foundational role. Adding self-efficacy and self-esteem in step 2 increased the explained variance to 58.7%, highlighting the critical contribution of these psychological factors. Self-efficacy (β = 0.695) was identified as the most significant predictor, underscoring its importance in understanding happiness among elders. These clarifications have been incorporated into the manuscript for greater clarity. | These changes are now reflected in lines 227–236 of the revised Results. |
| **Results** **Detailing Key Findings**: For the significant predictors, such as self-efficacy and self-esteem, a brief explanation on why they contribute so heavily to happiness in your population could provide a deeper understanding of your results. | We appreciate your insightful comment regarding the need to elaborate on the significant predictors of happiness, such as self-efficacy and self-esteem. In response, we have added an explanation in the Results section to clarify their roles. | These changes are now reflected in lines 242–247 of the revised Results. |
| **Discussion Causal Inferences:** The discussion seems to make causal interpretations from a cross-sectional study. Since your study is correlational, emphasize that while relationships were found, causality cannot be inferred. Adding a statement to this effect in the discussion and limitations will strengthen the scientific rigor of your work. | All the comments were made point-by-point and marked by track changes and highlighting in the revision file of the manuscript. |  |
| **Discussion Comparative Analysis**: Compare your findings more explicitly with studies in different cultural or geographical settings. You cite many studies from other countries (e.g., Turkey, Thailand), but a more nuanced comparison of how these findings align with or differ from your results will add depth. | All the comments were made point-by-point and marked by track changes and highlighting in the revision file of the manuscript. The results of this research compare with studies in Tehran, Iran, Hamadan, Iran. |  |
| **Discussion Mechanisms of Influence**: In lines 266-268, you mention that increasing self-efficacy and self-esteem can enhance happiness. Discuss potential mechanisms for this influence. For instance, is it because of improved coping strategies, greater social participation, or reduced psychological distress? | All the comments were made point-by-point and marked by track changes and highlighting in the revision file of the manuscript. It was added. |  |
| **Discussion Interventions**: In lines 305-307, be more specific about the types of community-based programs you recommend. For example, "Programs incorporating cognitive-behavioral therapy or mindfulness training could effectively boost self-efficacy and self-esteem." | All the comments were made point-by-point and marked by track changes and highlighting in the revision file of the manuscript. It was added. |  |
| **Limitations Expanded Limitations:** You mention that the study’s cross-sectional design limits your ability to draw causal conclusions. It would be beneficial to mention other limitations, such as reliance on self-reported measures, which can introduce bias. Also, acknowledge any potential biases in sampling (e.g., if the study underrepresents a certain subgroup of the elderly population in Sarab). | These limitations have been added. |  |
| **Limitations Generalizability**: The generalizability of your results outside the Iranian context could be addressed. You may want to elaborate on how cultural differences might affect self-efficacy, self-esteem, and happiness relationships in other contexts. | All the comments were made point-by-point and marked by track changes and highlighting in the revision file of the manuscript. It was corrected. |  |
| **Conclusion Action-Oriented Suggestions:** Your conclusion should not only summarize the findings but also provide clearer actionable recommendations for healthcare providers and policymakers. Rather than just suggesting “preventive and supportive actions,” you could specify programs that could be implemented, such as "targeted cognitive-behavioral programs aimed at boosting self-efficacy and emotional regulation in older adults." | Thank you for this insightful suggestion. We have revised the conclusion to not only summarize our findings but also to include more specific, actionable recommendations for healthcare providers and policymakers. | This can be found in lines 342–360 of the revised Conclusion. |
| **Language and Formatting**  **Clarity and Consistency:** The manuscript can be improved by revising some repetitive phrases and ensuring consistency in terms (e.g., consistently using either "older adults" or "elderly" rather than switching between them). Also, ensure that all abbreviations are defined the first time they are used.  **Grammar and Flow:** In some sections, the flow of ideas is somewhat disrupted by repetitive transitions (e.g., lines 235-236). Try to vary sentence structure and reduce redundancy to improve readability. | All the comments were made point-by-point and marked by track changes and highlighting in the revision file of the manuscript. It was added. |  |
| **Novelty:**Emphasize the unique contributions of your research. For example, highlight how your study provides new insights into an under-researched population (Iranian elderly) in the context of self-efficacy, self-esteem, and happiness. Stress this in both the introduction and conclusion to reinforce the study’s value. | In conclusion and discussion sections, new insights have be added |  |

With the changes outlined in this letter, we are confident that the manuscript  is scientifically richer and of higher quality.

We thank you and the reviewers for your patience and thorough review of this manuscript.

Sincerely,

Khalil Maleki Chollou
